# Multi-scale optical scanning holography for robust manipulation detection in satellite images

**DOI:** 10.1038/s41598-026-67323-1

**Published:** 2026-08-26

**Authors:** Mayada Khairy

**Affiliations:** Computers and Systems Department, Electronics Research Institute, Cairo, Egypt

**Keywords:** Optical Scanning Holography, Satellite Image, Deepfake Detection, Representation, Frequency-Domain Analysis Networks, Engineering, Mathematics and computing, Optics and photonics

## Abstract

Recent progress in generative AI has made it increasingly easy to produce highly realistic satellite imagery. While this advancement enables a wide range of beneficial applications, it also raises serious concerns regarding data reliability, misinformation, and security in critical remote sensing scenarios. Detecting manipulated satellite images remains a challenging task, as they lack human-related cues and instead exhibit complex spatial structures, where artifacts are often subtle and widely distributed. This paper introduces a novel multi-scale framework based on Optical Scanning Holography (OSH) to address this challenge. OSH is employed as a physics-inspired transformation that maps input images into a more informative feature space, where spatial, frequency, and phase characteristics are jointly represented within a unified domain. To further improve feature extraction, OSH is applied at multiple scales to generate complementary representations, which are combined into a multi-channel input. A convolutional neural network (CNN) is then utilized to learn discriminative patterns from this enhanced feature space. Experimental results on a large-scale dataset of real and AI-generated images demonstrate the effectiveness of the proposed framework, achieving an accuracy of 99.31%.

## Introduction

Satellite imagery has become a key component in modern decision-making, supporting a wide range of applications such as environmental monitoring, urban planning, disaster response, and military surveillance^1,2^. Because of its perceived neutrality and objectivity, this type of data is often taken at face value. However, even minor manipulations can lead to misleading interpretations and, in some cases, serious real-world consequences.

In recent years, advances in deep learning—particularly in generative models such as GANs and diffusion-based methods—have made it possible to produce satellite images that are visually indistinguishable from real ones^3–7^. While these developments open the door to many useful applications, they also introduce new risks. Synthetic images, despite their realism, can be used to spread misinformation or distort analysis, raising growing concerns around data authenticity and security^8,9^.

Detecting manipulated satellite imagery is far from straightforward. Unlike facial deepfakes, where biological or behavioral cues such as eye-blinking patterns can sometimes be used^10^, satellite images lack human-centered features altogether. Instead, they are defined by complex spatial layouts, textures, and large-scale contextual relationships. As a result, manipulation artifacts tend to be subtle and spread across the image, making them difficult to identify using standard techniques^8,9^.

Most existing approaches rely on convolutional neural networks (CNNs) operating in the spatial domain. These models are effective at capturing local texture patterns but often struggle with broader structural inconsistencies. Transformer-based methods attempt to overcome this limitation by modeling long-range dependencies, but they typically come with higher computational costs and require large-scale datasets^11,12^. At the same time, much of the research has focused on refining model architectures, with less attention given to how the input itself is represented.

Another important limitation is the heavy reliance on spatial information. In many cases, manipulation artifacts are not clearly visible in the spatial domain but become more apparent when viewed in the frequency domain. Irregular spectral patterns and hidden inconsistencies can often be detected in this space^13–15^. Despite this, frequency-aware representations are still relatively underexplored in the context of satellite image forensics.

To address these challenges, this work takes a different direction by focusing on improving the input representation rather than increasing model complexity. Specifically, we adopt Optical Scanning Holography (OSH) as a physics-inspired transformation that maps the input image into a richer and more structured representation space. This transformation captures spatial, frequency, and phase information in a unified manner.

The OSH process applies a modulation mechanism governed by a frequency-domain transfer function, creating a structured interaction between spatial and spectral components. This interaction enables the identification of subtle inconsistencies and hidden patterns that are otherwise difficult to observe, effectively transforming the image into a space where manipulation artifacts become more distinguishable.

Although OSH has been widely used in optical imaging and signal processing, its role in this context is different. Instead of being applied for reconstruction or encryption, it is utilized as a feature representation tool for detecting manipulated satellite imagery.

Building on this idea, the proposed approach applies OSH at multiple scales, where each scale captures complementary frequency characteristics at different levels of detail. These representations are combined to form a multi-channel input, enabling the model to utilize both spatial and frequency-domain information more effectively.

Overall, this work demonstrates that OSH can act as an effective representation mechanism, improving the visibility of structural and frequency-domain inconsistencies and enhancing the detection of manipulated satellite images. The remainder of this paper is organized as follows. Section "Related work" reviews the related work on manipulated satellite image detection, including spatial, frequency-domain, and deep learning-based approaches. Section "Methodology" presents the proposed methodology, including the Optical Scanning Holography (OSH) transformation, multi-scale feature representation, and CNN-based classification framework. Section "Evaluation and results" describes the experimental setup, dataset, and evaluation metrics, followed by a detailed analysis of the results. Section "Discussion" discusses the effectiveness of the proposed approach, including ablation studies and comparison with existing methods. Finally, Section "Conclusion" concludes the paper and outlines directions for future work.

## Related work

Research on detecting manipulated satellite imagery has attracted considerable attention in recent years, mainly due to the increased complexity of this problem compared to traditional deepfake detection. Unlike facial data, satellite images lack human-centered or physiological cues, requiring detection methods to depend more on structural patterns and contextual relationships within the scene.

Existing studies^9^ indicate that synthetic satellite images frequently exhibit large-scale irregularities, such as repeated terrain patterns, unnatural transitions, or inconsistent shadow behavior. These artifacts are generally not localized; instead, they tend to appear across the image, making them difficult to isolate using conventional spatial analysis techniques. This challenge is further influenced by the high resolution and visual complexity of remote sensing data, where forged images may appear nearly indistinguishable from real ones.

### Spatial-domain and transformer-based methods

Convolutional Neural Networks (CNNs) are widely used in image forensics because of their capability to capture fine-grained texture details and low-level artifacts. Bayar and Stamm^16^ were among the first to show that a constrained convolutional layer can learn manipulation-agnostic forensic features directly from data, and subsequent studies have demonstrated that CNN-based models can identify subtle traces left by generative processes^4,5,17^. Verdoliva^18^ provides a broader overview of how such CNN-based forensic tools fit within the wider media-forensics landscape. However, their dependence on local receptive fields restricts their capacity to capture long-range dependencies. Consequently, they may fail to capture broader inconsistencies, such as duplicated regions or unusual spatial arrangements extending across larger areas of the image.

To overcome this limitation, Vision Transformers (ViTs) have been proposed as an alternative framework. Through the use of self-attention mechanisms, these models are able to capture global relationships and long-range interactions more effectively^11^. Recent studies show that transformer-based approaches may outperform CNNs in detecting AI-generated satellite imagery, especially in identifying repetitive structures and scene-level inconsistencies^9^. However, these advantages often come with increased computational requirements and a stronger dependence on large-scale datasets.

### Frequency-domain forensic methods

Beyond spatial-domain analysis, a substantial body of work has shown that generative models leave characteristic traces in the frequency domain, and several distinct transform families have been explored for this purpose.

**Fourier-based (FFT/DFT) residual analysis.** Durall et al.^13,19^ demonstrated that the up-sampling operations common in GAN and diffusion decoders systematically distort the spectral properties of generated images, producing a characteristic decay in high-frequency energy that differs from real images, and proposed detecting forgeries by averaging the amplitude spectrum obtained via the Discrete Fourier Transform. Frank et al.^15^ further showed that these Fourier-domain artifacts persist across a wide range of GAN architectures, making DFT-based residual features a popular general-purpose forensic cue.

**DCT-based forgery detection.** The Discrete Cosine Transform has also been used directly as a forensic feature space rather than only as a pre-processing step. Qian et al.^20^ proposed F3-Net, which decomposes images into frequency bands via DCT and mines both frequency-aware decomposed components and local frequency statistics for forgery detection. This confirms that DCT coefficients can carry complementary, and at times more localized, forensic information than raw Fourier amplitude spectra.

**Wavelet-based forensics.** A separate line of work argues that global frequency transforms such as FFT and DCT discard spatial localization, which limits their ability to indicate *where* an artifact occurs. Wolter et al.^21^ showed that wavelet-packet representations, which preserve a degree of spatial locality alongside frequency decomposition, reveal statistically significant coefficient differences between real and GAN-generated images. More recently, Baru et al.^22^ combined wavelet decomposition with large pretrained vision-language backbones (Wavelet-CLIP) to improve cross-generator generalization, at the cost of additional computational overhead introduced by the decomposition and reconstruction steps.

**Learned and generalizable frequency-domain detectors.** More recent work has moved beyond fixed transform families toward learning frequency-aware representations end-to-end. Tan et al.^23^ proposed FreqNet, which embeds high-frequency representations directly into the classifier to improve cross-generator generalization, while Essa^24^ combined vision-transformer and MLP-Mixer feature fusion to strengthen sensitivity to fine-grained generative artifacts. Juefei-Xu et al.^14^ survey this broader family of frequency-based deepfake detectors and note that, while such methods generalize well to unseen synthesis techniques, they remain comparatively sensitive to common image degradations such as compression and resizing—a limitation that motivates representations, like the one proposed here, that are less reliant on a single fixed transform.

**Frequency-domain forensics for remote sensing imagery.** Compared to the face-forgery literature, frequency-aware detection specifically for satellite and remote-sensing imagery remains comparatively limited. Qi et al.^25^ proposed SFNet, which explicitly fuses independent spatial- and frequency-domain feature extractors for remote sensing forgery detection, motivated by the observation that the nature of forgery artifacts in remote sensing imagery varies substantially with geographic terrain and land-cover type, and that relying on a single visual cue limits generalization across diverse generative sources.

To situate OSH within this landscape, it is useful to clarify the physical distinction between it and transforms such as FFT and DCT. FFT and DCT are purely mathematical basis transforms: they decompose an image into linear combinations of fixed, image-independent basis functions (complex exponentials for FFT, real-valued cosines for DCT), and the resulting coefficients describe global frequency content with no direct correspondence to spatial position. OSH, by contrast, applies a physically motivated modulation defined by a quadratic-phase optical transfer function (Eq. 1), which is mathematically equivalent to simulating coherent optical propagation, such as Fresnel diffraction or lens defocus, rather than projecting the image onto an orthogonal basis. This modulation redistributes spatial-frequency energy back into the spatial domain as an interference-like pattern that couples local structure with global spectral content, rather than producing a set of decoupled frequency coefficients.

This property is particularly relevant to satellite imagery. Unlike facial deepfakes, where forgery traces tend to be localized to specific facial regions, manipulation artifacts in satellite scenes are typically diffuse and manifest as subtle deviations in large-scale, quasi-periodic structures such as terrain texture, road grids, or building layouts^9^. Because the OSH modulation acts globally across the spectrum while remaining sensitive to how that spectral content is spatially organized, it is well suited to exposing this kind of broadly distributed structural irregularity, a combination of properties that a purely local representation (wavelet) or a purely global and spatially decoupled one (FFT/DCT) does not offer on its own.

Taken together, this body of work indicates that global transforms such as FFT and DCT are effective at exposing spectral irregularities but process frequency components largely independently of spatial location and of each other’s phase relationships, while wavelet-based methods partially restore spatial locality at the cost of the unified phase-frequency coupling that a physical imaging system would otherwise encode. To the best of our knowledge, none of these representations combine spatial, spectral, and phase information within a single physically-grounded transfer function in the way OSH does. This gap motivates the use of Optical Scanning Holography in this work, whose physical formulation and implications for manipulation detection are discussed in further detail in Section "Optical Scanning Holography (OSH) Transformation".

## Methodology

The proposed framework is designed to improve the detection of manipulated satellite images by going beyond standard spatial representations. Rather than focusing solely on modifying model architectures, the approach places emphasis on how the input itself is represented. In this context, OSH is employed as a physics-inspired transformation that maps the image into a more informative feature space, where spatial and frequency characteristics are jointly represented.

In contrast to conventional frequency-domain techniques based on Fourier analysis, OSH does not simply separate frequency components. Instead, it generates a holographic representation through a modulation process that captures spatial structure, frequency distribution, and phase information in a unified form^26,27^. This richer representation facilitates the identification of subtle inconsistencies introduced by generative models—patterns that are often difficult to detect when relying solely on spatial information^13,14^.

Based on this idea, the proposed framework introduces a multi-scale OSH strategy. Rather than using a single transformation, the input image is processed at multiple scales, enabling different aspects of the spectral content to be captured at varying levels of detail. These complementary representations offer a more comprehensive description of the image, which in turn supports more reliable discrimination between authentic and manipulated samples.

The resulting representations are combined into a multi-channel input and then fed into a convolutional neural network (CNN). Rather than relying on hand-crafted features or shallow models, as in traditional forensic pipelines, the CNN learns hierarchical features directly from the enriched input space, capturing both fine-grained patterns and broader structural relationships without explicit feature engineering.

It is important to note that, although high-frequency components often contain useful cues for detecting manipulation^5,15,28^, relying solely on frequency analysis is not sufficient. Complex distortions introduced by modern generative models typically span multiple domains. For this reason, OSH is utilized as a projection-based representation mechanism that integrates spatial, frequency, and phase information, resulting in a more discriminative feature space.

Multi-scale representations have been widely shown to improve robustness and generalization in deep learning systems^29,30^. Within this framework, each OSH scale contributes distinct spectral characteristics, and their combination enhances the overall expressiveness of the input. This multi-channel design allows the network to better capture subtle artifacts that might otherwise remain unnoticed.

**Fig. 1:**
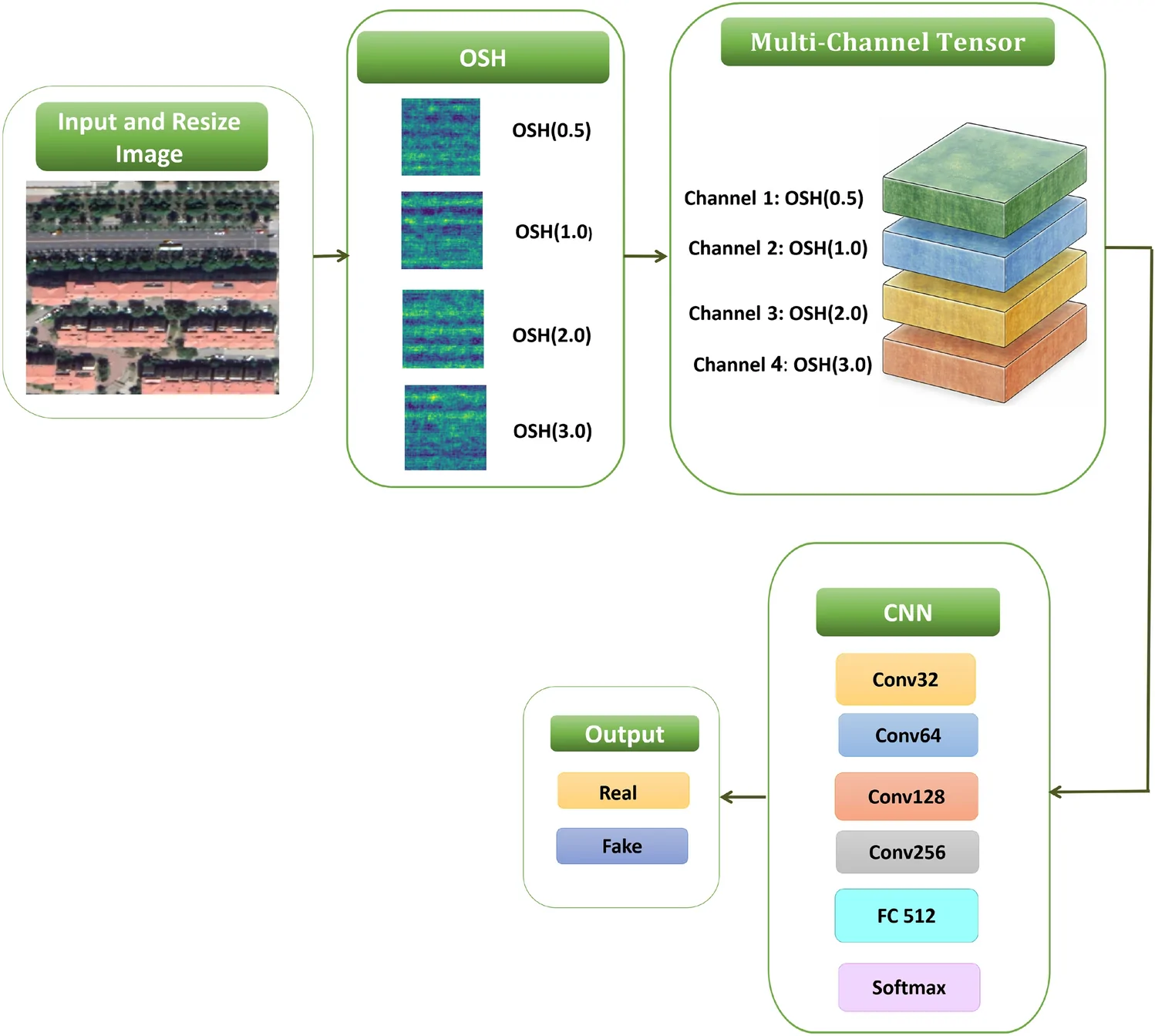
Overview of the proposed multi-scale OSH-based framework for detecting manipulation in satellite images.

As illustrated in Fig. 1, the overall pipeline consists of four main stages:

(i) image preprocessing,
(ii) multi-scale OSH transformation,
(iii) multi-channel feature construction,
(iv) CNN-based classification.

By integrating multi-scale holographic representations with deep learning, the proposed framework enhances both the robustness and the discriminative capability of manipulated image detection.

### Optical scanning holography (OSH) transformation

OSH is employed in this work as a transformation that maps the input image into a richer and more structured representation space. Instead of treating spatial and frequency information as separate components, OSH establishes a controlled interaction between them through a modulation process defined by an Optical Transfer Function (OTF)^27,31^. This enables different aspects of the image to be encoded jointly, resulting in a more informative representation.

**Fig. 2:**
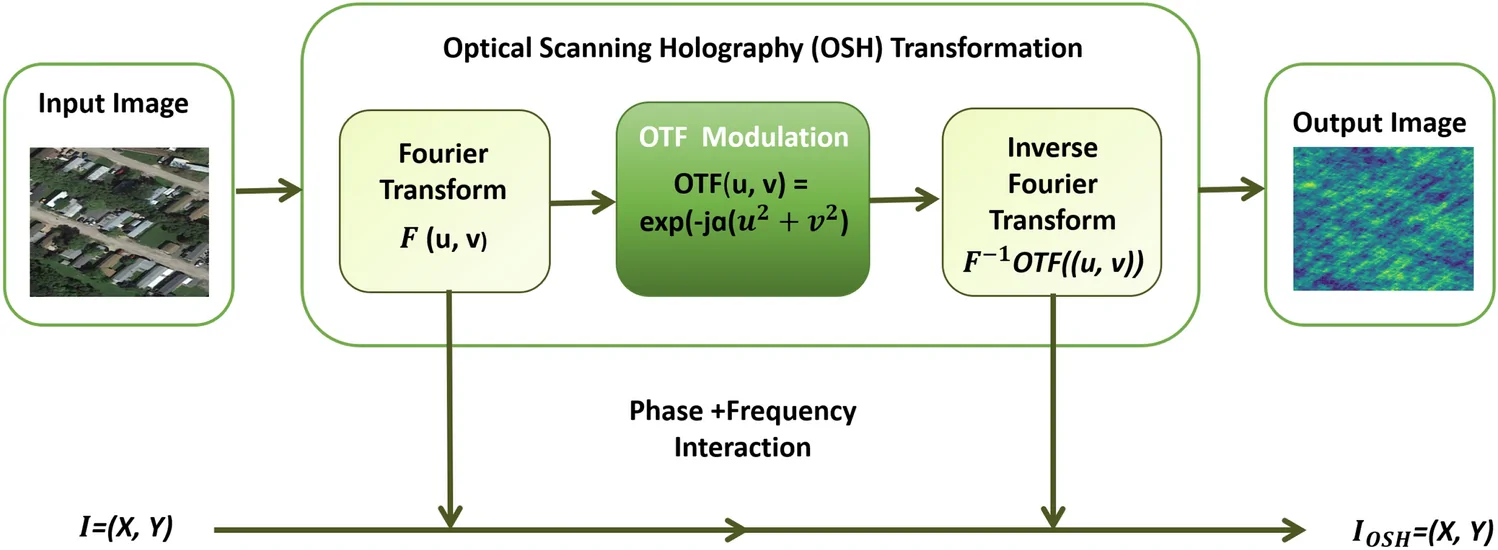
Optical Scanning Holography (OSH) transformation pipeline.

In contrast to standard frequency-domain methods based on Fourier decomposition^32^, where frequency components are processed independently, OSH forms a unified representation that also incorporates phase information. This proves particularly useful for highlighting subtle inconsistencies that are often difficult to observe in the spatial domain alone^13,14^. An overview of the transformation process is presented in Fig. 2.

The transformation is defined in the frequency domain using the Optical Transfer Function (OTF):

1 $$\begin{aligned} OTF(u,v) = \exp \left( -j\alpha (u^2 + v^2)\right) \end{aligned}$$

where (*u*, *v*) denote the spatial frequency coordinates and *α* controls the frequency response.

Physically, *α* plays the role of a chirp-rate (curvature) coefficient of the quadratic phase term in the OTF, analogous to the *1/(λ z)* term that governs the curvature of a Fresnel diffraction kernel at propagation distance *z* for wavelength *λ* in a physical optical scanning system^27,31^. A small value of *α* corresponds to a shallow phase curvature, so the OTF varies slowly across (*u*, *v*) and the modulation primarily redistributes energy among coarse, low-frequency spatial structures. As *α* increases, the phase term curves more steeply, and the OTF oscillates more rapidly across the frequency plane, so the modulation increasingly folds finer, high-frequency detail into the resulting interference pattern. In this sense, *α* behaves as a scale parameter that determines which band of spatial frequencies is most strongly redistributed by the holographic modulation, in the same way that propagation distance controls which spatial scales are emphasized in a physical Fresnel hologram.

This interpretation motivates the specific set of values used in this work, $\alpha \in \{0.5, 1.0, 2.0, 3.0\}$. Rather than an arbitrary choice, the values are selected to span, in an approximately geometrically increasing progression, a range from shallow phase curvature (emphasizing coarse, large-scale structural regularities such as terrain layout) to steep phase curvature (emphasizing fine-grained, high-frequency texture). Combining representations from across this range in the multi-channel tensor of Eq. (6) is intended to provide the network with complementary views of the same scene at different effective spatial-frequency scales, which is consistent with the ablation results in Table 2, where accuracy improves monotonically as additional *α* scales are added (Section "Effect of OSH representation").

To further quantify the individual contribution of each scale, a sensitivity analysis was conducted in which a separate single-channel model was trained using only one OSH(*α*) representation at a time, keeping the training/validation subsets, model initialization, and all other hyperparameters identical across runs so that *α* was the only variable being changed. Table 1 reports the resulting accuracy, precision, recall, and F1-score for each individual *α* value.

**Table 1:** Sensitivity of detection performance to the OSH scale parameter *α*, evaluated using a single-channel OSH representation for each value in isolation.

*α*	Accuracy (%)	Precision	Recall	F1-score
0.5	93.38	0.9417	0.9875	0.9641
1.0	94.38	0.9542	0.9847	0.9692
2.0	93.25	0.9359	0.9931	0.9636
**3.0**	**95.12**	**0.9570**	0.9903	**0.9734**

As shown in Table 1, each individual *α* value already yields a strong single-channel classifier, with accuracy ranging from 93.25% (*α =2.0*) to 95.12% (*α =3.0*). The highest-curvature setting, *α =3.0*, achieves the best individual performance, consistent with the intuition that fine-grained, high-frequency texture is particularly informative for distinguishing generative upsampling artifacts. Notably, the relationship between *α* and accuracy is not strictly monotonic: *α =2.0* performs marginally below both *α =0.5* and *α =1.0* despite corresponding to a steeper phase curvature. This indicates that different *α* values emphasize partially distinct and non-redundant spatial-frequency bands rather than a single scale that is uniformly best. Critically, every individual *α* value in Table 1 underperforms the combined four-channel configuration (OSH4, 99.31% in Table 2) by a wide margin, confirming that the performance of the proposed framework arises primarily from the complementarity between scales rather than from any single optimal *α*.

The computation of the OSH representation follows a sequence of operations. First, the input image is transformed into the frequency domain:

2 $$\begin{aligned} F_I(u,v) = \mathcal {F}\{I(x,y)\} \end{aligned}$$

The resulting spectrum is then modulated using the OTF:

3 $$\begin{aligned} F_H(u,v) = F_I(u,v) \cdot OTF(u,v) \end{aligned}$$

After modulation, the signal is converted back into the spatial domain:

4 $$\begin{aligned} \tilde{I}_{OSH}(x,y) = \mathcal {F}^{-1}\{F_H(u,v)\} \end{aligned}$$

Finally, the magnitude of the reconstructed signal is extracted:

5 $$\begin{aligned} I_{OSH}(x,y) = |\tilde{I}_{OSH}(x,y)| \end{aligned}$$

Unlike conventional approaches that isolate frequency components, this process redistributes them through a structured interaction between spatial and spectral domains^27,31^. As a result, the transformed representation highlights patterns that are otherwise difficult to detect.

In practice, this representation facilitates the identification of subtle artifacts and inconsistencies introduced by generative models. When combined with deep learning, it provides a more reliable basis for distinguishing between authentic and manipulated satellite images^5,15,28^.

**Fig. 3:**
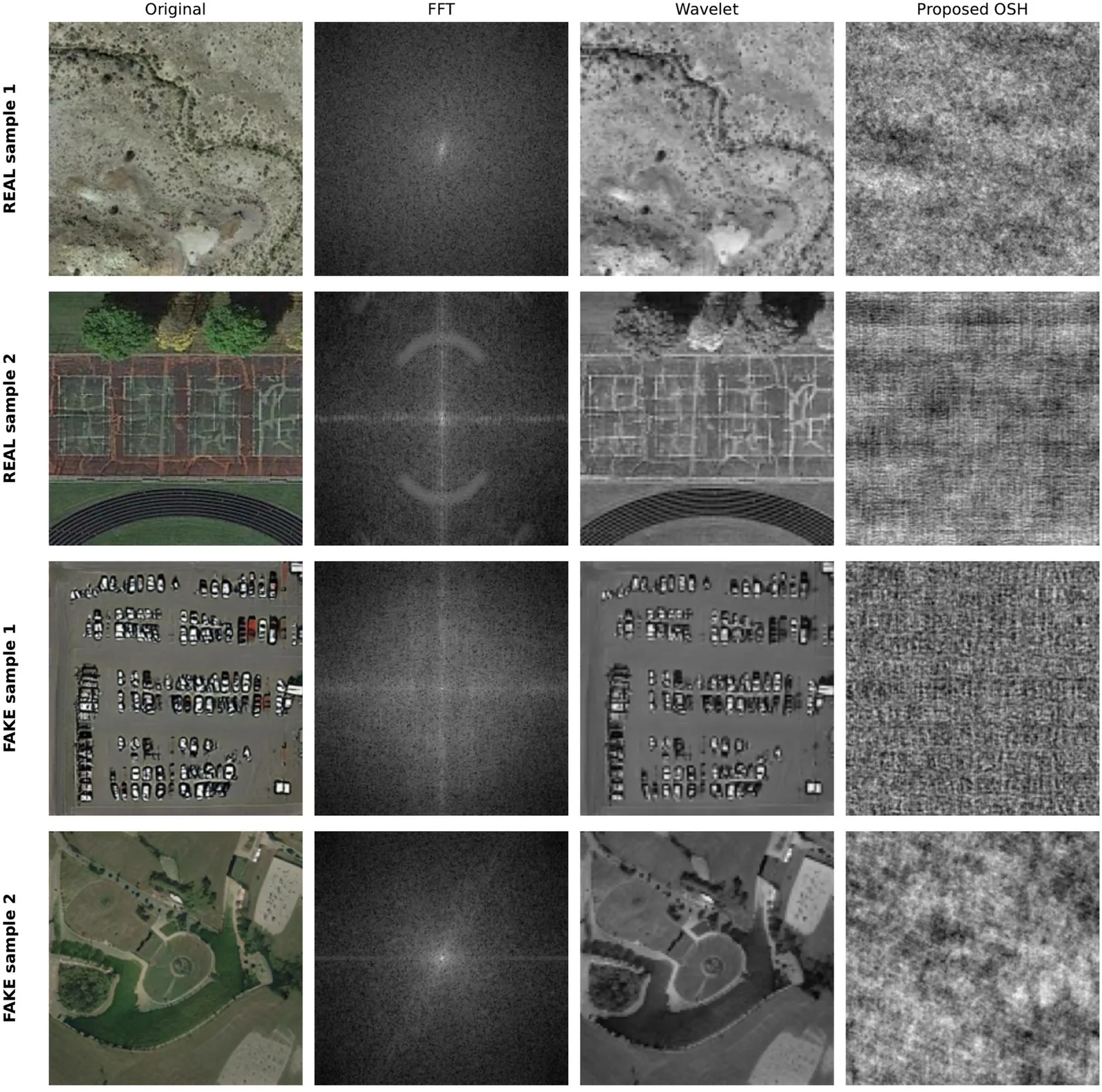
Comparison of FFT, wavelet, and the proposed OSH representations for authentic and manipulated satellite samples. Unlike FFT and wavelet, OSH reveals regular, grid-like textures in manipulated samples that are absent in authentic samples, highlighting its discriminative value.

To empirically illustrate the physical distinction between OSH and conventional frequency-domain transforms discussed above, Fig. 3 presents a qualitative comparison of the standard FFT magnitude spectrum, the Haar wavelet LL sub-band, and the proposed OSH representation for two representative authentic and two manipulated satellite samples. While the FFT and wavelet representations of authentic and manipulated samples remain visually similar and offer limited discriminative cues, the OSH representation reveals a marked difference between the two classes: authentic samples exhibit irregular, non-repetitive micro-textures, whereas manipulated samples display more regular, grid-like periodic patterns. This visual evidence directly supports the physical rationale for adopting OSH and clarifies why it is not merely an alternative frequency-domain filter, but a phase-preserving representation that exposes structural regularities inaccessible to conventional magnitude-only transforms.

### Multi-channel feature construction

To make better use of frequency-aware information, the proposed approach generates multiple Optical Scanning Holography (OSH) representations at different scales. Instead of relying on a single transformation, these representations are combined to form a unified multi-channel input. Unlike conventional methods that include the original spatial image alongside transformed features, the formulation adopted here relies entirely on OSH-based representations.

The resulting multi-channel input is defined as:

6 $$\begin{aligned} X = \left[ I^{(0.5)}_{OSH}(x,y),\; I^{(1.0)}_{OSH}(x,y),\; I^{(2.0)}_{OSH}(x,y),\; I^{(3.0)}_{OSH}(x,y)\right] \end{aligned}$$

More generally, the tensor can be written as:

7 $$\begin{aligned} X(x,y,c) = I^{(\alpha _c)}_{OSH}(x,y), \quad c = 1,2,3,4 \end{aligned}$$

where $I^{(\alpha )}_{OSH}(x,y)$ represents the OSH-transformed image at scale *α*, and *c* denotes the channel index.

By combining multiple OSH projections, the framework effectively constructs a feature space that spans different representation domains. Each channel highlights distinct frequency-domain and phase-related properties, allowing the model to capture patterns that may not be apparent when using a single transformation. This multi-scale perspective makes it easier to identify subtle inconsistencies that are often associated with manipulated content^5,15,28^.

Rather than treating the channels as simple stacked inputs, the design can be viewed as a structured form of feature augmentation. Each component contributes complementary information, which enhances both the expressive capacity of the representation and the robustness of the model.

Interestingly, experimental results suggest that OSH-based representations alone are sufficiently informative for the task. Including the original spatial image does not necessarily improve performance and may introduce redundancy, as some spatial features are already implicitly encoded within the transformed representations.

**Fig. 4:**
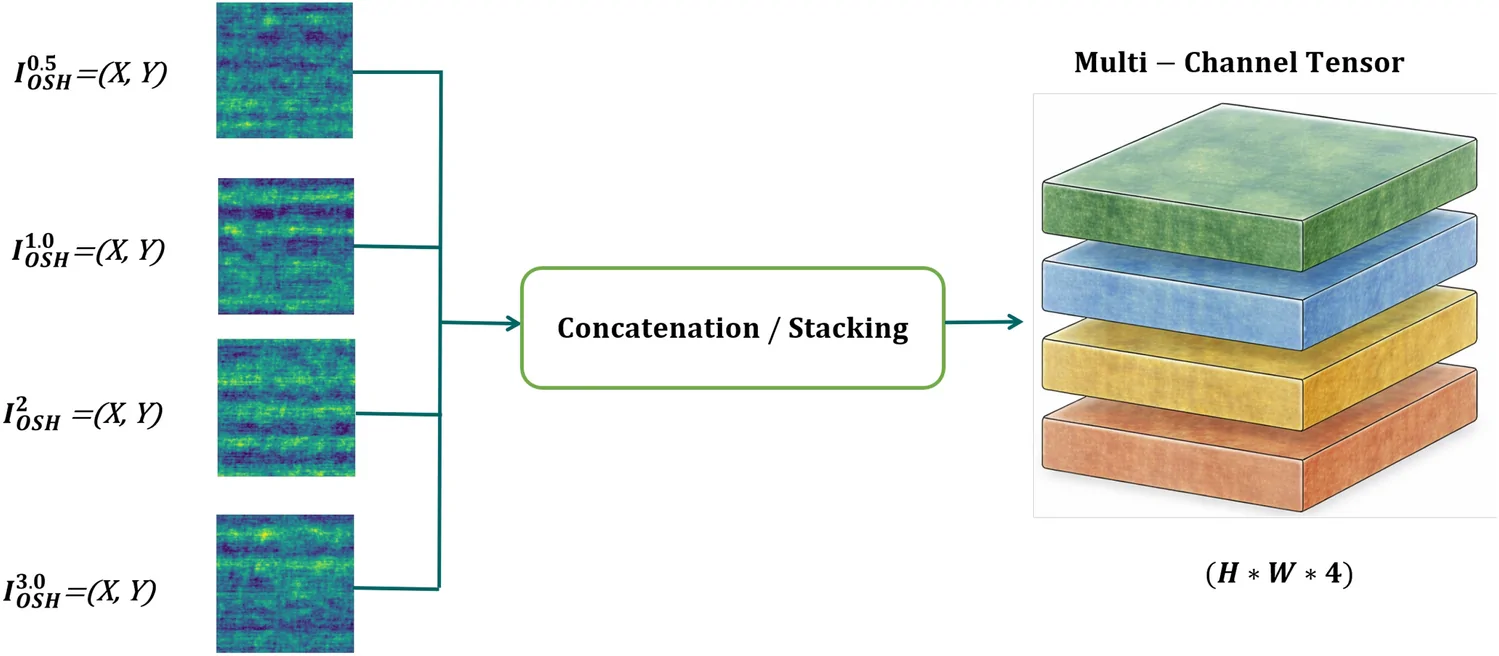
Multi-channel OSH-based feature construction where multiple OSH representations at different scales are combined to form a unified tensor.

Multi-channel fusion has been widely adopted in deep learning to improve representation learning and classification performance^30,33^. However, unlike standard RGB inputs or conventional feature stacking, the channels in this framework are derived from physics-informed transformations. This leads to a more task-specific and discriminative representation that is better suited for detecting manipulated satellite imagery (Fig 4).

### CNN architecture and training strategy

A convolutional neural network (CNN) is used to learn hierarchical feature representations from the multi-channel input tensor *X* of size $4 \times 224 \times 224$. CNNs have proven to be effective in capturing spatial patterns and extracting discriminative features in a wide range of visual recognition tasks^30,33^.

In contrast to conventional CNN pipelines that operate on standard RGB inputs, the proposed model takes a multi-domain input. This input combines spatial, frequency, and phase-aware information, allowing the network to benefit from complementary cues that are not available in a single representation domain.

The feature extraction process can be expressed as:

8 $$\begin{aligned} y = f_{\text {CNN}}(X) \end{aligned}$$

The network is composed of four convolutional blocks with channel sizes of 32, 64, 128, and 256, respectively. Each block includes a convolutional layer followed by batch normalization, a ReLU activation, and a max-pooling operation. This structure enables the model to gradually move from low-level feature extraction to more abstract representations, while also reducing the spatial dimensions of the feature maps.

Batch normalization is applied after each convolutional layer to stabilize training and improve convergence. By reducing internal covariate shift, it helps the model train more efficiently.

After the convolutional stages, the resulting feature maps are flattened and passed through fully connected layers. The first fully connected layer consists of 512 neurons, followed by a ReLU activation and a dropout layer with a rate of 0.4 to mitigate overfitting^34^. The final layer outputs two neurons corresponding to the target classes (authentic vs. manipulated).

The final prediction is obtained using the Softmax function:

9 $$\begin{aligned} \hat{y} = \text {Softmax}(Wz + b) \end{aligned}$$

The strength of this architecture lies in its ability to learn from a structured multi-channel input rather than relying solely on raw spatial information. By integrating spatial, frequency, and phase-related features, the model is better equipped to identify subtle inconsistencies that may not be captured by traditional spatial-domain approaches.

**Fig. 5:**
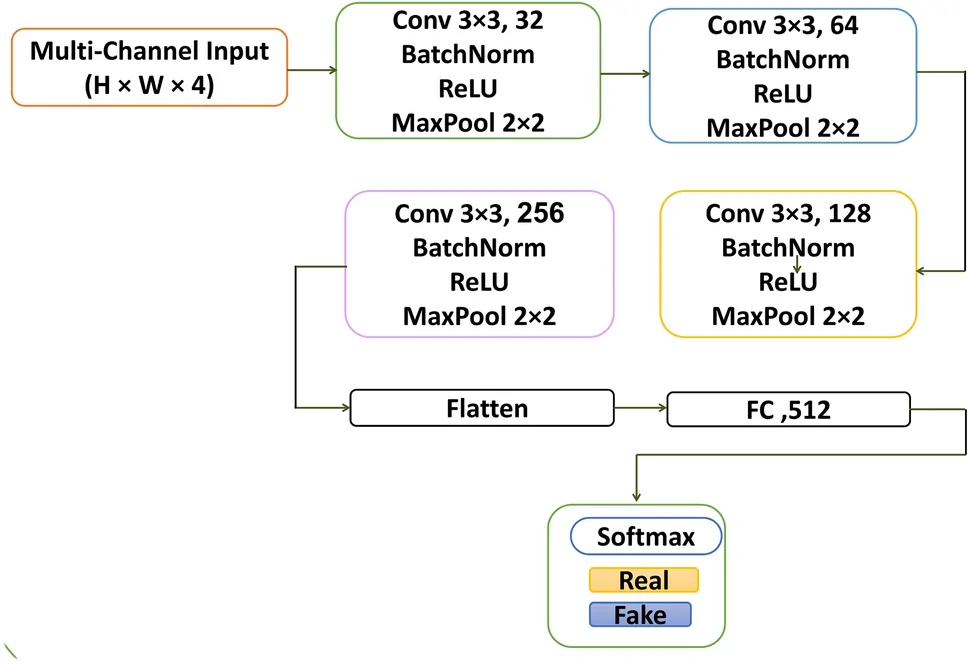
Proposed CNN architecture for multi-channel OSH-based feature extraction and manipulated image detection.

An overview of the proposed CNN architecture is shown in Fig. 5.

#### Training Strategy

The model is trained for the task of manipulated image detection using the cross-entropy loss function with label smoothing:

10 $$\begin{aligned} \mathcal {L} = - \sum _{i=1}^{N} y_i^{\text {LS}} \log (\hat{y}_i) \end{aligned}$$

where $y_i^{\text {LS}}$ denotes the label-smoothed target distribution obtained with a smoothing factor of 0.05, which slightly softens the hard 0/1 targets to reduce overconfidence while preserving a sharp decision boundary between the real and manipulated classes.

Model parameters are optimized using the AdamW optimizer^35^, a variant of Adam^36^ with decoupled weight decay, with an initial learning rate of $10^{-4}$ and a weight decay of $3\times 10^{-4}$. This choice provides a good balance between convergence speed and generalization, while also helping to stabilize the training process.

To further improve training dynamics, a learning rate scheduler (ReduceLROnPlateau) is used. Instead of keeping the learning rate fixed, it is adjusted based on validation performance, allowing the model to converge more smoothly when progress slows down.

In addition, early stopping is applied as a regularization strategy. Training is halted when no improvement is observed on the validation set for a predefined number of epochs, reducing the risk of overfitting.

An exponential moving average (EMA) of the model weights is maintained throughout training, with a decay rate of 0.999, and validation is performed using the EMA weights rather than the raw model weights at each epoch; the checkpoint used for final evaluation corresponds to the EMA weights achieving the highest validation accuracy. At validation and test time, predictions are further obtained using flip-based test-time augmentation (TTA), where the softmax outputs of the original and horizontally-flipped input are averaged before the final class decision. Both EMA and TTA are applied for evaluation purposes only and do not introduce additional learnable parameters into the model.

To increase robustness, simple data augmentation techniques are incorporated during training. In particular, random horizontal flipping is applied to introduce variability in the input data, which helps the model generalize better to unseen samples.

Algorithm 1 summarizes the end-to-end pipeline described in this section, from the input image to the final authenticity prediction. Steps 2–6, corresponding to the OSH transformation (Eqs. (1)–(5)), are entirely deterministic and require no learnable parameters, whereas step 8 (the CNN classifier) is the only trainable component of the framework, consistent with the computational complexity analysis in Section Computational complexity analysis.

**Algorithm 1:**
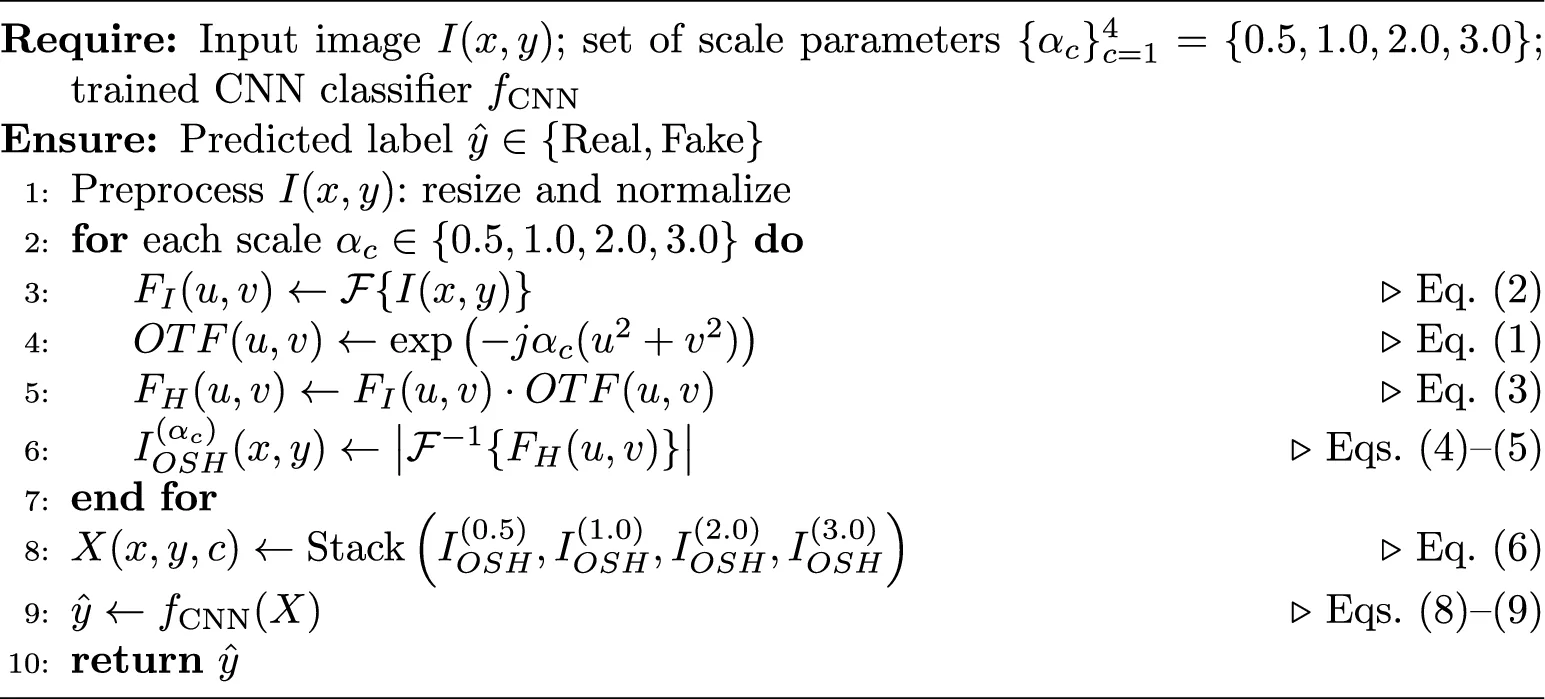
Multi-scale OSH-based manipulation detection.

## Evaluation and results

### Dataset description

To evaluate the proposed framework in a reliable and comprehensive way, a large and diverse satellite imagery dataset is utilized. The dataset contains both authentic and AI-generated images, enabling the model to be evaluated across a range of manipulation scenarios.

The data are collected from two publicly available benchmarks: the DM-AER dataset^37^ and the FSI dataset^38^, both widely used in satellite image forgery detection research^9,39^. Combining these datasets increases variability in terms of image content, manipulation types, and generative techniques.

In total, the dataset consists of more than 300,000 images, including both real and manipulated samples. The DM-AER dataset provides the majority of the data, featuring high-resolution images along with synthetic samples generated using advanced models such as StyleGAN2. The FSI dataset further contributes to diversity by introducing manipulations generated through CycleGAN-based methods.

All samples are labeled as either authentic or manipulated. The dataset is divided into training, validation, and testing subsets following a standard protocol to ensure fair evaluation.

To reduce bias, the distribution of authentic and manipulated samples is maintained in a balanced manner across all subsets. This contributes to improved generalization and supports stable model performance during both training and evaluation.

Using multiple datasets exposes the model to a broader range of artifacts and manipulation patterns. Compared to relying on a single data source, this configuration improves robustness and generalization.

Importantly, the dataset is suitable for evaluating frequency-aware approaches. It includes subtle spectral irregularities and structural inconsistencies introduced by generative models, which are consistent with the objectives of the proposed OSH-based representation in highlighting such patterns.

Overall, the scale, diversity, and complexity of the dataset provide a strong foundation for evaluating the effectiveness of the proposed method.

### Overall model performance

The proposed OSH-based framework is evaluated using a held-out test set of 10,000 satellite images, comprising both authentic and AI-generated samples.

The model reaches an accuracy of 99.31%, with a precision of 0.9871, recall of 0.9998, and an F1-score of 0.9935. These values reflect a strong capability in differentiating between real and manipulated images.

**Fig. 6:**
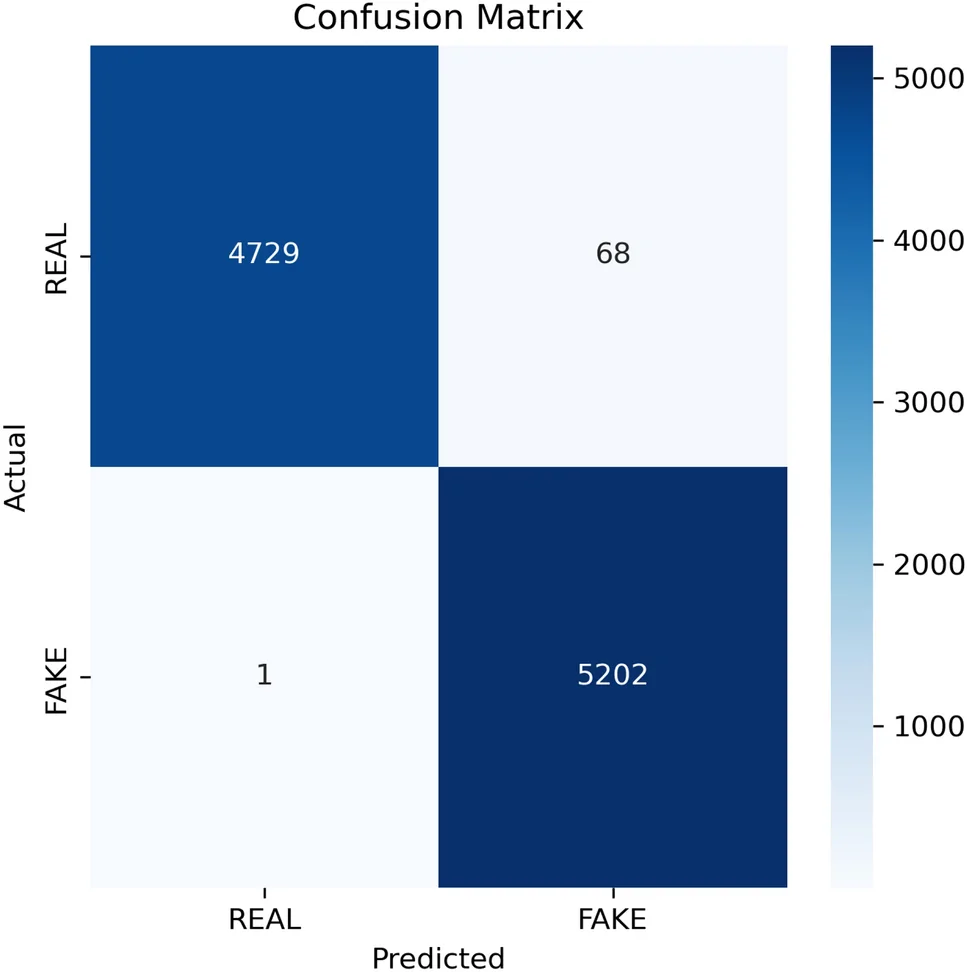
Confusion matrix of the proposed OSH-based model evaluated on the test set.

A clearer picture of the model’s predictions can be seen from the confusion matrix in Fig. 6, which presents the distribution of true positives, true negatives, false positives, and false negatives.

From the test set, the model correctly classifies 5202 manipulated images and 4729 authentic ones, with only 1 false negative and 68 false positives. This indicates that most samples are classified correctly, with a very limited number of errors.

Only one manipulated image is incorrectly labeled as authentic, corresponding to a false negative rate of approximately 0.02% and a recall of 99.98%. This aspect is particularly relevant in practical scenarios, where failing to detect a manipulated sample can lead to serious consequences.

In contrast, 68 authentic images are misclassified as manipulated, resulting in a false positive rate of about 1.42%. Although this introduces some false alarms, it reflects a slightly conservative tendency, where the model leans toward detecting potential manipulations.

This difference between false negatives and false positives indicates that the model places greater emphasis on identifying manipulated samples, which is often preferred in security-sensitive applications.

Overall, the results show that the proposed framework achieves high accuracy and robustness, while maintaining strong sensitivity to manipulated content and a controlled level of false detections.

### Training behavior analysis

To better understand how the model learns over time, both the training loss and validation accuracy were tracked across epochs.

**Fig. 7:**
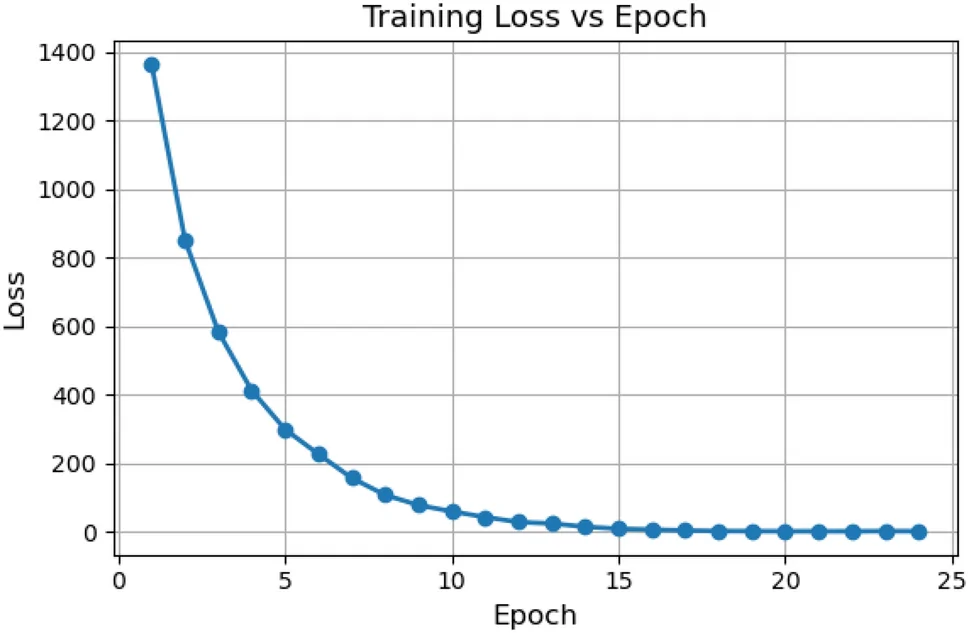
Training loss over epochs showing the convergence behavior of the proposed model.

As shown in Fig. 7, the training loss drops rapidly during the early stages of training and then gradually levels off as it approaches very small values. This pattern suggests that the model is able to learn efficiently from the data and reach convergence without unnecessary delay.

**Fig. 8:**
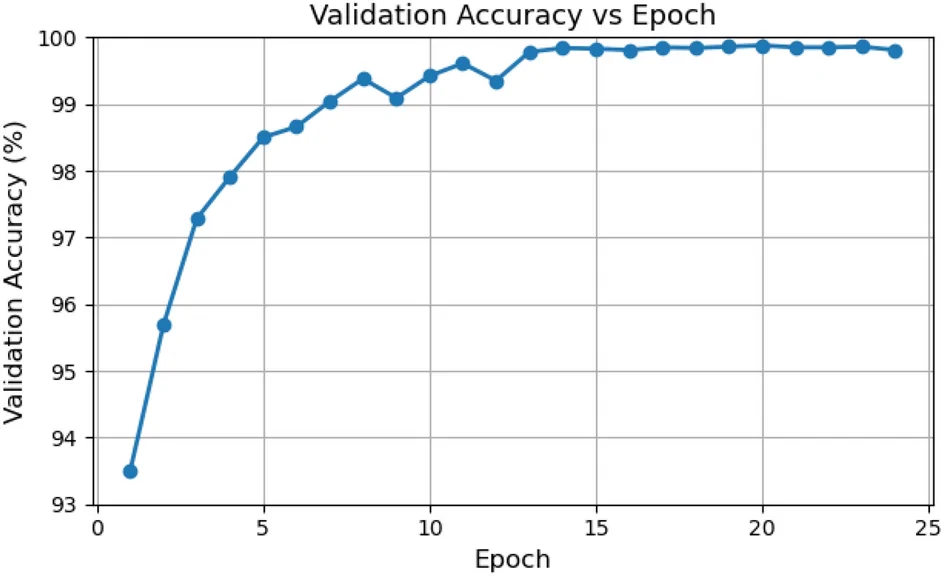
Validation accuracy over epochs illustrating consistent performance improvements.

A similar trend can be observed in Fig. 8, where the validation accuracy improves steadily from 93.50% in the first epoch to approximately 99.88%. Notably, the model achieves strong performance early on and maintains it throughout the later stages of training.

While small fluctuations are visible in the validation curve, they are minor and expected in stochastic optimization settings. Importantly, these variations do not point to any instability in the training process.

Another key observation is the close alignment between training and validation behavior. The absence of a noticeable gap between the two indicates that the model generalizes well and is not significantly affected by overfitting.

Overall, the training curves reflect stable convergence, efficient learning dynamics, and strong generalization capability, supporting the reliability of the proposed framework.

### Explainability analysis using grad-CAM

To provide deeper insight into the model’s decision-making process, Grad-CAM visualization is employed to analyze the spatial attention of the network for both manipulated and authentic satellite images. These visualizations make it possible to determine which regions of the image contribute most to the final prediction.

**Fig. 9:**
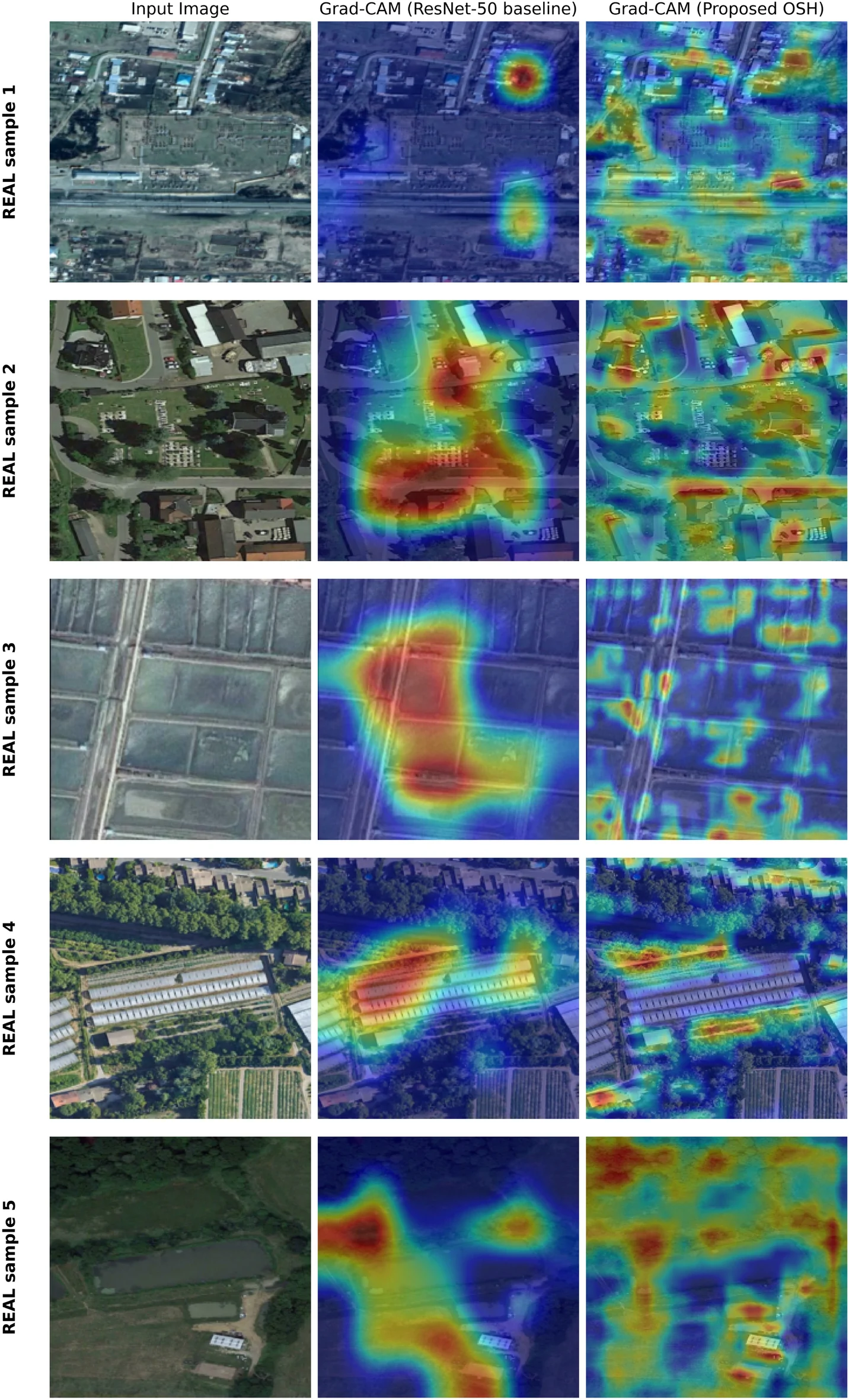
Grad-CAM comparison between the spatial-domain ResNet-50 baseline and the proposed OSH-based model for five representative authentic (REAL) satellite image samples. For each sample, the input image (left) is shown alongside the Grad-CAM overlay produced by the ResNet-50 baseline (middle) and by the proposed OSH-based model (right).

**Fig. 10:**
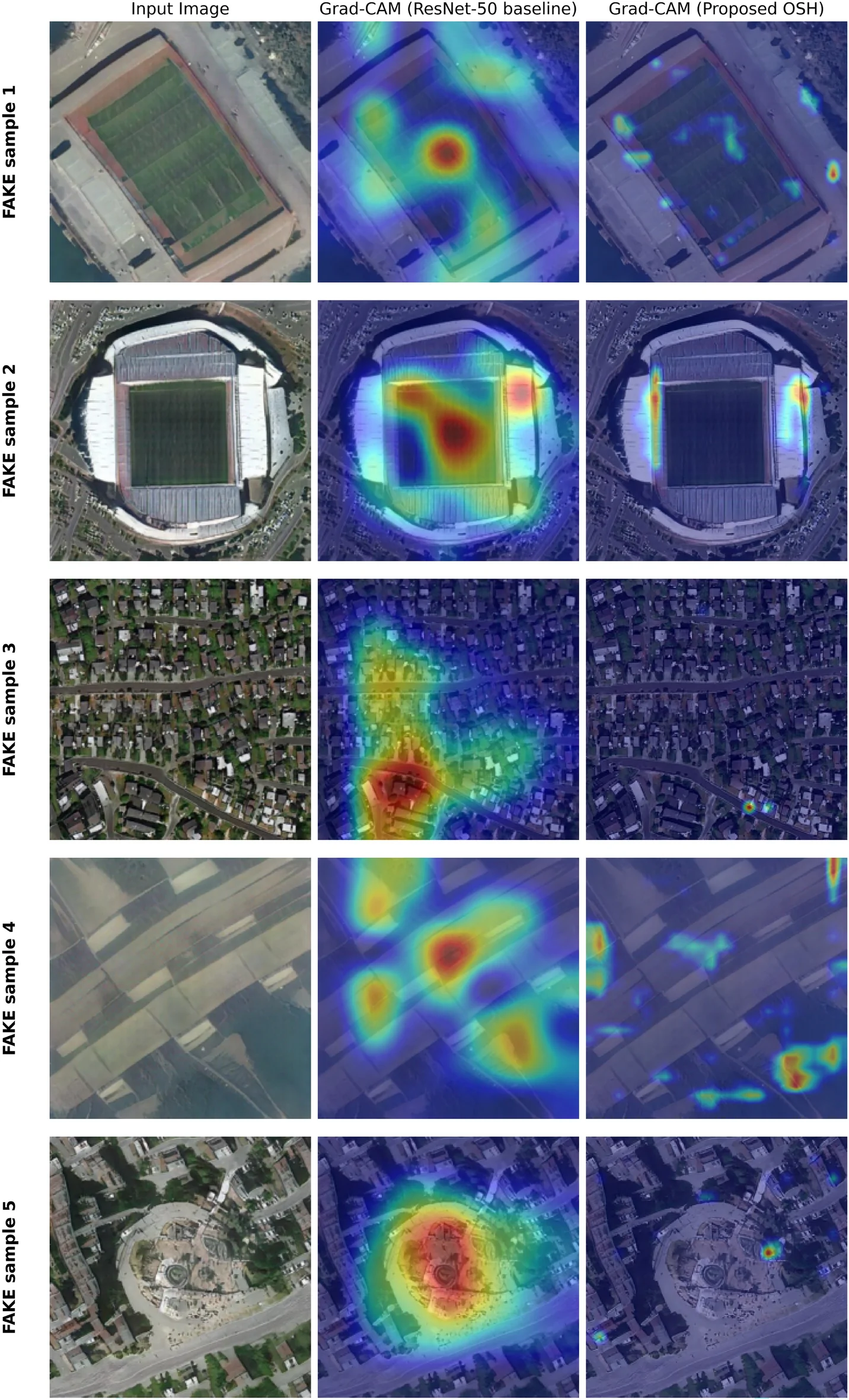
Grad-CAM comparison between the spatial-domain ResNet-50 baseline and the proposed OSH-based model for five representative manipulated (FAKE) satellite image samples. For each sample, the input image (left) is shown alongside the Grad-CAM overlay produced by the ResNet-50 baseline (middle) and by the proposed OSH-based model (right).

Figures 9 and 10 present representative examples from five authentic and five manipulated samples, respectively. For each sample, the input image is shown alongside the Grad-CAM overlay obtained from a spatial-domain ResNet-50 baseline and the Grad-CAM overlay obtained from the proposed OSH-based model, allowing a direct qualitative comparison between the two attention behaviors across a broader range of scenes.

For manipulated images, the Grad-CAM visualizations reveal noticeably different attention patterns between the two models. While the ResNet-50 baseline frequently responds to broad semantic regions or large salient structures, the proposed OSH-based model often emphasizes different regions that are consistent with its frequency-aware representation. These differences suggest that the proposed representation encourages the network to exploit complementary structural and frequency-domain cues rather than relying solely on conventional spatial appearance.

For authentic images, both models attend to meaningful scene structures; however, their attention distributions differ qualitatively. The proposed OSH-based model exhibits attention patterns that are consistent with the multi-scale frequency-aware representation, whereas the ResNet-50 baseline primarily reflects spatial-domain characteristics.

Overall, this qualitative comparison complements the quantitative results reported in Table 4 and provides qualitative evidence that the proposed OSH representation enables the network to exploit image characteristics that differ from those learned by a purely spatial-domain CNN.

### Effect of OSH representation

To examine the contribution of the proposed OSH-based representation, an ablation study is conducted by gradually increasing the number of OSH-transformed inputs.

**Table 2:** Effect of increasing the number of OSH representations.

Representation	Accuracy (%)	Precision	Recall	F1-score
OSH1	91.73	0.9100	0.9050	0.9075
OSH2	94.83	0.9450	0.9400	0.9425
OSH3	97.48	0.9720	0.9680	0.9700
**OSH4**	**99.31**	**0.9871**	**0.9998**	**0.9935**

Each configuration represents a different number of OSH representations. Specifically, OSH1 applies a single transformation, whereas OSH2, OSH3, and OSH4 incorporate two, three, and four representations, respectively.

As shown in Table 2, performance improves steadily as additional OSH representations are introduced. The accuracy increases from 91.73% with a single input to 99.31% when four representations are used. This consistent improvement indicates that each additional scale contributes complementary information.

A similar trend is observed for precision, recall, and F1-score, indicating that the multi-scale design improves the model’s ability to detect manipulated samples more reliably. In particular, the recall reaches 0.9998 in the OSH4 configuration, meaning that nearly all manipulated images are correctly identified.

These results emphasize the importance of multi-scale representations. By combining information from different scales, the model captures subtle artifacts that may not be apparent when relying on a single transformation.

### Comparison with raw image input

To further evaluate the effectiveness of the proposed representation, a comparison is carried out between raw image input and OSH-based configurations.

**Table 3:** Comparison between raw image and OSH-based representations.

Input Type	Accuracy (%)	Precision	Recall	F1-score
Image only	88.16	0.8810	0.8720	0.8760
Image + OSH4	99.00	0.9890	0.9910	0.9900
**OSH4**	**99.31**	**0.9871**	**0.9998**	**0.9935**

As presented in Table 3, the OSH-based representation outperforms the raw image input, with accuracy increasing from 88.16% to 99.31%.

Combining the original image with OSH representations improves performance compared to using the image alone. However, this combination does not surpass the performance obtained when relying exclusively on OSH features. This indicates that the transformed representations already capture the most relevant information required for the task.

In contrast, incorporating the raw spatial input may introduce redundant or less informative features, which contribute minimally to the final prediction.

Overall, the results confirm the effectiveness of the proposed OSH transformation. The observed performance gains primarily stem from the multi-scale holographic representation, which offers a more informative and task-specific feature space for detecting manipulated satellite images.

### Comparison with State-of-the-Art methods

To provide a more comprehensive evaluation, the proposed OSH-based framework is compared with several established deep learning approaches reported in the literature.

Convolutional Neural Networks (CNNs), such as ResNet-50, are widely used for learning spatial features^30^. In parallel, Vision Transformer (ViT) models have demonstrated strong performance in capturing long-range dependencies in visual data^11^. In the context of satellite image manipulation detection, recent studies^9^ indicate that CNN-based models typically achieve an accuracy of approximately 87.02%, while transformer-based approaches such as ViT can reach around 95.11%.

**Table 4:** Comparison with existing deep learning approaches.

Method	Architecture Type	Accuracy (%)
ResNet-50^30^	CNN	87.02
ViT^11^	Transformer	95.11
**Proposed OSH4**	CNN + OSH	**99.31**

As shown in Table 4, the proposed method achieves a clear improvement over both CNN-based and transformer-based approaches.

Beyond generic CNN and transformer baselines, it is also informative to situate the proposed framework relative to domain-specific frequency-based forensic methods and to methods specifically designed for remote-sensing forgery detection. Table 5 summarizes representative methods from each transform family discussed in Section "Frequency-domain forensic methods" (FFT-residual, DCT-based, wavelet-based, and remote-sensing-specific hybrid approaches), together with their transform domain, target imagery, and reported performance as stated in the original publications.

**Table 5:** Landscape of domain-specific frequency-based forgery detection methods relative to the proposed framework. Because these methods were evaluated on different benchmarks, generative sources, and forgery types (mostly face forgery) than the DM-AER and FSI satellite datasets used in this work, the reported values are not directly comparable under a shared protocol and are included for qualitative context only; a numerically direct comparison is left as future work.

Method	Transform Domain	Target Imagery	Reported Performance	Benchmark
Durall et al.^13,19^	FFT amplitude spectrum	GAN-generated (general)	Characteristic high-frequency spectral decay used as a detection cue	Multiple GAN datasets
Frank et al.^15^	Fourier-domain artifacts	GAN-generated (general)	Confirms persistent Fourier-domain artifacts across GAN architectures	Multiple GAN datasets
Qian et al. (F3-Net)^20^	DCT frequency bands	Face forgery	Outperforms spatial-domain SOTA baselines, especially on low-quality compressed media	FaceForensics++
Wolter et al.^21^	Wavelet-packet decomposition	GAN-generated (general)	Statistically significant coefficient differences vs. real images	Multiple GAN datasets
Baru et al. (Wavelet-CLIP)^22^	Wavelet + vision-language features	Cross-generator images	Improved cross-generator generalization, at added computational cost	Multiple generators
Geo-DefakeHop^40^	Subspace learning (non-deep)	Satellite imagery	F1-score > 95% under common distortions	UW Fake Satellite Image
SFNet^25^	Spatial-frequency fusion	Satellite imagery	81.82% accuracy, 76.97% F1-score	ISPRS-FD
**Proposed (OSH4)**	Spatial + frequency + phase (OSH)	Satellite imagery	**99.31% accuracy, 0.9935 F1-score**	DM-AER + FSI

As shown in Table 5, prior frequency-domain forensic methods each exploit a single transform family (FFT, DCT, or wavelet) and are predominantly developed and evaluated on face-forgery benchmarks rather than satellite imagery. Among the two methods specifically designed for remote-sensing forgery detection, Geo-DefakeHop^40^ is a lightweight, non-deep-learning detector based on parallel subspace learning, while SFNet^25^ fuses independent spatial- and frequency-domain feature extractors and outperforms other frequency-domain and remote-sensing-specific hybrid baselines evaluated in the same study (e.g., FcaNet, FreqNet, FSLNet). Nonetheless, the reported performance of these specialized methods illustrates that hybrid spatial-frequency and subspace-learning approaches are an active and competitive direction in remote-sensing forgery detection, reinforcing the motivation for representation-centric methods such as the one proposed here rather than relying solely on generic spatial-domain architectures.

This improvement can be attributed primarily to the OSH-based representation, which enriches the feature space by integrating spatial, frequency, and phase-related information within a unified framework^26,27^. Unlike conventional approaches that focus mainly on increasing model complexity, the proposed method emphasizes improving the representation of the input data, enabling more effective detection of subtle inconsistencies associated with manipulated content.

Although frequency-domain methods have been explored in image forensics^13–15^, their effectiveness in satellite imagery remains limited^39^. This is mainly due to the complex spatial structures and large-scale contextual dependencies present in satellite images, where manipulation artifacts are often subtle and widely distributed.

Most existing approaches rely on Fourier-based representations^32^, where frequency components are treated independently, without explicitly modeling their interaction with spatial information. As a result, such methods may struggle to capture the complex structural inconsistencies that characterize manipulated satellite scenes.

In contrast, the proposed OSH-based framework captures the interaction between spatial, frequency, and phase components in a structured manner^26,27^, making subtle manipulation artifacts more distinguishable, particularly in complex satellite scenes.

Overall, the results suggest that improving input representation can be more effective than simply increasing model complexity, as reflected in the strong performance achieved without relying on heavily parameterized architectures.

### Computational complexity analysis

Since satellite image processing pipelines often operate under near-real-time constraints, the computational cost of the full proposed framework is an important complement to the accuracy results reported above. Table 6 summarizes the trainable parameter count, floating-point operations (FLOPs), end-to-end inference time, throughput, and peak GPU memory usage of the complete pipeline, encompassing the multi-scale OSH preprocessing (four FFT/IFFT passes across all *α* scales), the multi-channel tensor construction, and the CNN forward pass. FLOPs were computed using the THOP profiling library; inference time is reported as the mean ± standard deviation over repeated end-to-end pipeline executions on a raw input image, and peak GPU memory reflects the maximum memory allocated during the full pipeline execution.

**Table 6:** Computational complexity of the proposed framework (end-to-end, including multi-scale OSH preprocessing and CNN inference).

Metric	Value
Input size	$4 \times 224 \times 224$
Trainable parameters	26.081 M
FLOPs	1.578 GFLOPs
End-to-end inference time	1.163 ± 0.044 ms/image
Throughput	860.13 FPS
Peak GPU memory	552.13 MB
Hardware	NVIDIA GeForce RTX 4090 Laptop GPU

The reported inference time in Table 6 represents the end-to-end execution time of the complete proposed pipeline, measured from a raw input image through the multi-scale OSH transformation (four FFT/IFFT passes, one per *α* scale), the multi-channel tensor construction, and the CNN forward pass to the final authenticity prediction. The end-to-end latency of 1.163 ms per image, corresponding to a throughput of 860.13 FPS, confirms that the full framework is well suited to near-real-time deployment under satellite image processing constraints. It is worth noting that the OSH preprocessing is a deterministic, parameter-free operation whose cost scales with image resolution and the number of *α* scales rather than with model parameters; this distinction is preserved in the FLOPs count, which reflects the full pipeline computation including all FFT/IFFT operations.

## Discussion

The experimental results provide strong evidence for the effectiveness of the proposed OSH-based framework in detecting manipulated satellite imagery. The observed performance improvements are primarily attributed to the enhanced feature representation introduced by the multi-scale OSH transformation, rather than to changes in model complexity.

In contrast to conventional approaches that rely mainly on spatial information, the proposed method represents the input within a multi-domain space that integrates spatial, frequency, and phase components. This enriched representation facilitates the identification of subtle inconsistencies introduced by generative models—patterns that are often difficult to detect when relying solely on spatial features.

The ablation study further reinforces this observation. The monotonic accuracy improvement from 91.73% (OSH1) to 99.31% (OSH4) is consistent with the physical interpretation of the *α* parameter: each additional scale emphasizes a distinct spatial-frequency band, so their combination provides the network with complementary views of the scene that no single scale can offer alone.

The comparison with raw image input reveals a further insight: OSH representations alone outperform both the image-only and image+OSH configurations. This suggests that the OSH transformation already encodes the spatial cues present in the raw image—redistributing them into a more structured interference pattern—so that adding the original spatial input introduces redundancy rather than new discriminative information.

The confusion matrix analysis also provides insight into the model’s behavior. The extremely low false negative rate (0.02%) indicates that the model is highly effective at detecting manipulated images. In practical applications—particularly in security-sensitive contexts—this aspect is often more critical than minimizing false positives. The slightly higher false positive rate reflects a conservative tendency, where uncertain cases are more likely to be flagged rather than overlooked.

The training behavior analysis further indicates stable convergence: the gradual reduction in training loss, together with the steady improvement in validation accuracy and the absence of a large train–validation gap, suggests that the model generalizes well without clear signs of overfitting.

Taken together, the comparison against CNN- and transformer-based baselines (Table 4) shows that this improvement is achieved without a deeper or more computationally intensive architecture, reinforcing the central claim of this work: how the input is represented can matter as much as, or more than, how large the model is.

It is also important to consider the conditions under which the proposed framework would be deployed in practice. Satellite platforms are subject to micro-vibrations from onboard mechanisms such as reaction wheels, solar-array drives, and cryocoolers, which can introduce sub-pixel jitter, motion blur, and localized spectral distortions in the acquired imagery. Because the proposed detector relies on frequency- and phase-domain patterns, such physical artifacts could, in principle, interact with the OSH-transformed representation and affect detection robustness under real imaging conditions. Explicitly modeling this kind of platform-induced degradation during training, or introducing a dedicated compensation stage, is a promising direction for improving the practical robustness of the framework and is noted as a limitation below. In this regard, active compensation strategies developed for related satellite subsystems are of particular interest: for instance, Zhu et al.^41^ recently proposed a CNN-LSTM-based compound control scheme that predicts micro-vibration signals for feedforward compensation in inter-satellite optical communication systems, substantially suppressing platform-induced jitter at the acquisition stage. Integrating a similar predictive compensation mechanism upstream of the proposed OSH-based pipeline, so that the input imagery is pre-stabilized before the OSH transformation is applied, is a promising direction for future work aimed at improving robustness under realistic in-orbit imaging conditions.

### Limitations

Several limitations of the present study should be acknowledged. First, the dataset used for training and evaluation, while large and diverse, is drawn from two benchmarks whose manipulated samples are generated primarily by StyleGAN2- and CycleGAN-based methods; performance on imagery produced by newer diffusion-based generators, which the manuscript identifies as an increasingly important source of synthetic satellite imagery, has not been separately evaluated and may differ from the reported results; prior work on natural-image forensics has shown that diffusion models leave forensic traces that differ from those of GANs^42^ and has proposed detectors, such as DIRE, specifically tailored to diffusion-generated content^43^, and adapting or evaluating such techniques for the satellite domain is left for future work. Second, the current evaluation does not account for platform-induced physical artifacts such as micro-vibration, sensor noise, or atmospheric distortion that are present in operational satellite imaging conditions, as discussed above; the reported accuracy therefore reflects performance under comparatively clean imaging conditions rather than an operational remote-sensing pipeline.

## Conclusion

This paper presented a multi-scale framework based on Optical Scanning Holography (OSH) for detecting manipulated satellite imagery, built around a multi-domain feature space that jointly encodes spatial, frequency, and phase-related characteristics rather than relying on spatial information alone.

The experimental results support this design choice: the proposed framework reaches 99.31% accuracy with a very high recall on manipulated samples, outperforming CNN- and transformer-based baselines; the ablation study shows accuracy improving consistently as more OSH scales are added; and OSH features alone outperform configurations that also include the raw image, indicating that the representation itself, rather than added model complexity, drives the observed gains.

Overall, these findings support the broader view that improving the input representation can be at least as effective as increasing model depth for this task, and they position the proposed framework as a practical option for security-sensitive remote sensing applications. Future work will extend this approach to other modalities, such as video-based manipulation detection, and explore its integration into real-time and multimodal monitoring systems. Open robustness questions—including performance on diffusion-generated forgeries and resilience to platform-induced imaging artifacts—are also targeted directions for future investigation.

## Data Availability

The datasets used in this study are publicly available at the following repositories: https://figshare.com/s/eeedcd150e759ef4353c and https://github.com/RijulGupta-DM/deepfake-satellite-images.

## Code availability

The training and evaluation code developed for this work is available at https://doi.org/10.5281/zenodo.21419445.

## Declarations

### Research involving human and/or animals

This study does not involve human participants or animals.
